# Identification of two internal signal peptide sequences: critical for classical swine fever virus non-structural protein 2 to trans-localize to the endoplasmic reticulum

**DOI:** 10.1186/1743-422X-8-236

**Published:** 2011-05-18

**Authors:** Kang-kang Guo, Qing-hai Tang, Yan-ming Zhang, Kai Kang, Lei He

**Affiliations:** 1 College of Veterinary Medicine, Northwest A & F University, Yangling, Shaanxi 712100, P.R.China; 2 State Key Laboratory of Veterinary Biotechnology, Harbin Veterinary Research Institute, Chinese Academy of Agricultural Sciences, Harbin, Heilongjiang 150001, P.R. China

## Abstract

**Background:**

The membrane topology and molecular mechanisms for endoplasmic reticulum (ER) localization of classical swine fever virus (CSFV) non-structural 2 (NS2) protien is unclear. We attempted to elucidate the subcellular localization, and the molecular mechanisms responsible for the localization of this protein in our study. The NS2 gene was amplified by reverse transcription polymerase chain reaction, with the transmembrane region and hydrophilicity of the NS2 protein was predicted by bioinformatics analysis. Twelve cDNAs of the NS2 gene were amplified by the PCR deletion method and cloned into a eukaryotic expression vector, which was transfected into a swine umbilical vein endothelial cell line (SUVEC). Subcellular localization of the NS2 protein was characterized by confocal microscopy, and western blots were carried out to analyze protein expression.

**Results:**

Our results showed that the -NH_2 _terminal of the CSFV NS2 protein was highly hydrophobic and the protein localized in the ER. At least four transmembrane regions and two internal signal peptide sequences (amino acids103-138 and 220-262) were identified and thought to be critical for its trans-localization to the ER.

**Conclusions:**

This is the first study to identify the internal signal peptide sequences of the CSFV NS2 protein and its subcellular localization, providing the foundation for further exploration of this protein's function of this protein and its role in CSFV pathogenesis.

## Background

Classical swine fever (CSF) is a highly contagious and often fatal disease of pigs and is classified by the World Organization for Animal Health (OIE) as a notifiable (previously List A) disease. The causative agent of CSF is classical swine fever virus (CSFV), a member of the *Pestivirus *genus within the *Flaviviridae *family of viruses, which also contains the genera *Flavivirus *and *Hepacivirus *(hepatitis C viruses, HCV)[1]. CSFV harbors a 12.3 kb positive-sense, single-stranded RNA genome that consists of a large open reading frame that encodes a polyprotein which is processed into 12 mature proteins, namely, N^pro^, C, E^rns^, E1, E2, p7, NS2, NS3, NS4A, NS4B, NS5A and NS5B [2-4].

In recent years, the nonstructural NS2 protein has been thought to be functional only as an NS2/NS3 auto-protease, which is essential for high productivity of CSFV *in vivo*. It was speculated that the N-terminal half of NS2 is highly hydrophobic, and that p7 protein may contain a signal sequence to direct the downstream NS2 protein to the membrane [3,5,6]. Our previous study demonstrated that CSFV NS2 was a hydrophobic protein and localized in the endoplasmic reticulum (ER) membrane, independently of CSFV p7 peptides. However, the membrane topology and molecular mechanism of ER localization of this protein remains unclear. The biofunction of a protein is always associated with it's subcellular localization. For instance, HCV NS2 protein, which shares great similarities with CSFV NS2 protein, localizes in the ER membrane and lead to ER stress [7,8]. Interestingly, our results indicated that CSFV NS2 protein contains two internal signal peptide sequences, which are critical for trans-localization to the ER, and this protein probably possesses at least four transmembrane regions. The findings are crucial for elucidating the function of CSFV NS2 protein, and also have potentially important implications for understanding the molecular mechanisms of pathogenesis for this economically important agricultural disease.

## Materials and methods

### Vectors and cell culture

The pEGFP-C1 eukaryotic expression vector was purchased from Clontech (USA) and competent *E. coli *DH5α cells, which were used for cloning, were purchased from Tiangen Biotech (China). The pEGFP-NS2 plasmid contained the full-length NS2 gene from the CSFV virulent train Shimen. The established swine umbilical vein endothelial cell line (SUVEC) was cultured as previously described [9].

### Antibodies and reagents

Mouse anti-GFP monoclonal antibody (mAb) and horseradish peroxidase-conjugated goat anti-mouse antibodies were purchased from Millipore (USA). The nuclear staining dye Hoechst 33342 and ER-Tracker™ Red probe were obtained from Invitrogen (USA)

### Plasmid construction and transfection

**Figure 1:**
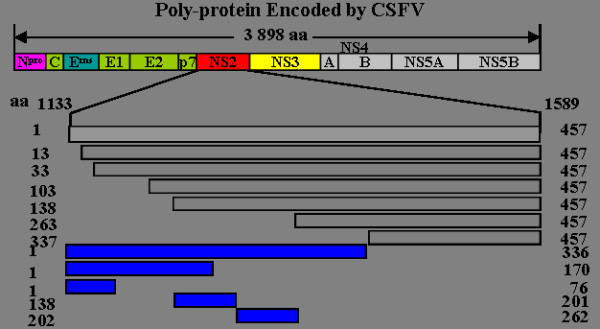
**Schematic representation of the CSFV NS2 deletion-mapping results**. NS2 is located at aa 1133-1589 in the CSFV strain Shimen polyprotein sequence. The numbers indicate the first and the last amino acids of each NS2 protein sequence used for the studies.

To investigate the internal signal sequences in the CSFV NS2 protein, the primers shown in Table 1 were designed according to the CSFV NS2 gene nucleotide sequences. All of the upstream primers contained a *Sal*Isite, and a *Bam*HIsite was incorporated into all of the downstream primers. Using these primers, 12 amino-terminal truncated polymerase chain reaction (PCR) products were obtained and the relative position of each amplified fragment is shown in Figure 1. PCR was carried out according to the following procedures (pEGFP-NS2 was used as a template): an initial denaturation step at 95°C for 5 min, followed by 35 cycles of 95°C for 30 sec, 60°C for 30 sec, 72°C for 1.5 min, and a final extension step at 72°C for 10 min. The 12 PCR products obtained, designated as NS2/1-457, NS2/13-457, NS2/33-457, NS2/103-457, NS2/138-457, NS2/263-457, NS2/337-457, NS2/1-336, NS2/1-170, NS2/1-76, NS2/202-262 and NS2/138-201 were cloned into the *Sal*I/*Bam*HIsites of the expression vector pEGFP-C1 and the recombinant plasmids were identified and verified by enzyme digestion and sequencing. SUVEC were seeded into 15 mm^2 ^confocal dish (Costar, USA) 24 h before being transfected. When they reached 60-70% confluence, the cells were transfected with the 12 recombinant plasmids and the pEGFP-C1 control vector using the Lipofectamine 2000 transfection reagent, according to the manufacturer's instructions (Invitrogen, USA)

**Table 1:** Primers for construction of 12 subcloning of cDNA.

Primers and sites(amino acid site of NS2)	Primer sequences (5"-3")
Anti457	**CAT*GGATCC***TCTAAGCACCCAGCCAAGG
Anti336	**CAT*GGATCC***CTTCGGCATCCCATAAACC
Anti170	**CAT*GGATCC***TGGCAGTATGAGGATCAGGG
Anti76	**CAT*GGATCC***AATGTAGGTCCAGGTTAGCAACG
S1	**CAT*GTCGAC ***GGAAAGATAGATGGCGGTTG
S13	**CAT*GTCGAC ***ACCAGCTTTGACATCCAACTC
S33	**CAT*GTCGAC ***AAGAGAGATCCGACTACTGTCCC
S103	**CAT*GTCGAC ***AAGGGAATAGGTGAGTTGGATTTAC
S138	**CAT*GTCGAC ***AATCTGGACATAGCCGGATTG
S263	**CAT*GTCGAC ***AAGAAGATCATAGATGAAATAGCAGG
S337	**CAT*GTCGAC ***TTGGTTGGCTTAGTCAAGGC
Anti201	**CAT *GGATCC ***GTTTACCCTCTTAAAGTTGGTCTTCC
S202	**CAT *GTCGAC ***GACATATATGAAGTTGACCAAGCTG
Anti262	**CAT *GGATCC ***GTGGAGGTAGTAAGACACTTCAAATATC

A restriction site sequence was inserted in 5' of each primer. The underlined sequences were restriction sites.

### Western blot analysis

Protein expression was analyzed by western blot as reported previously. Briefly, whole cell extracts were prepared by washing the cells with PBS, harvesting them by scraping and then resuspending the cells in 1 mL of PBS. Following centrifugation, the cells were resuspended in cell lysis buffer (50 mM Tris-HCl, 5 mM EDTA, 150 mM NaCl, 0.1% NP-40, 0.5% deoxycholic acid, 1 mM sodium orthovanadate, 100 μg/mL PMSF and protease inhibitors) and centrifuged at 15,000 × g for 30 min at 4°C. Cell extracts were resolved by 12% sodium dodecyl sulfate-polyacrylamide gel electrophoresis (SDS-PAGE) and transferred to a PVDF membrane (Millipore, USA). The membrane was blocked overnight with 5% skimmed milk in TNT buffer (20 mM Tris-HCl [pH 7.5], 150 mM NaCl, and 0.05% Tween 20) and then incubated with mouse anti-GFP mAb for 2 h. Detection of primary antibodies was performed with horseradish peroxidase-conjugated goat anti-mouse antibody, as appropriate. The protein bands were visualized by enhanced chemiluminescence methods according to the manufacturer's instructions (Millipore).

### Fluorescence staining and confocal microscopy

To examine the expression and subcellular localization of CSFV NS2 protein, 48 h after transfection, cells were washed with Hank's balanced salt solution (HBSS) and incubated with Hoechst33342 at 37°C for 15 min. The cells were washed twice with HBSS and incubated with ER-Tracker™ Red probe (Invitrogen, USA) at 37°C for 30 min. Cells were again washed with HBSS and then visualized by laser confocal scanning microscopy (Model LSM510 META, Zeiss, Germany).

### Bioinformatics analysis

The amino acid sequences of the CSFV NS2 protein was analyzed by the DNAstar software and the online analysis tool available at http://us.expasy.org/tools.

## Results

### Construction of recombinant expressing plasmid

**Figure 2:**
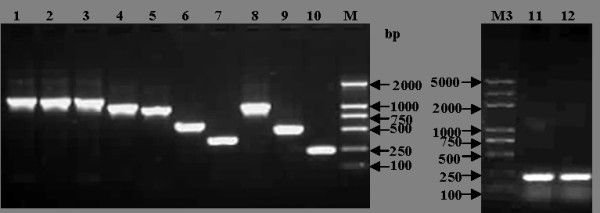
**PCR amplification of 12 subcoloning cDNA of CSFV NS2 gene**. M, DL 2000 marker; M3, Plus-DL2000 marker; 1~12, PCR products of 12 subcloning cDNA.

**Figure 3:**
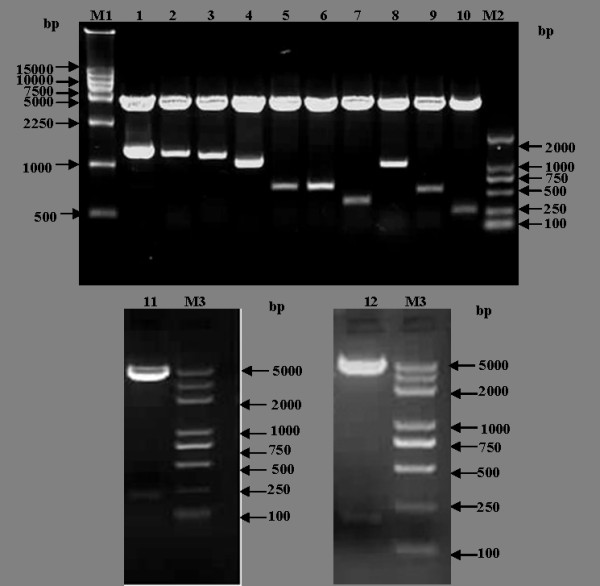
**Identificantion of the recombinant vector by enzyme digestion**. M1, DL15000 marker; M2, DL 2000 marker; M3, Plus DL 2000 marker; 1~12, Enzyme digestion of 12 recombinant vectors by SalIand BamHI.

Using specifically designed primers, 12 amino-terminal truncated fragments of the NS2 genes were amplified by PCR and the sizes of these amplified products were verified by electrophoresis (Figure 2). These PCR products were cloned into the expression vector pEGFP-C1, and the 12 recombinant plasmids were identified and verified by SalI/BamHI enzyme digestion (Figure 3) and sequence analysis.

### Expression and subcellular localization of CSFV NS2 protein

**Figure 4:**
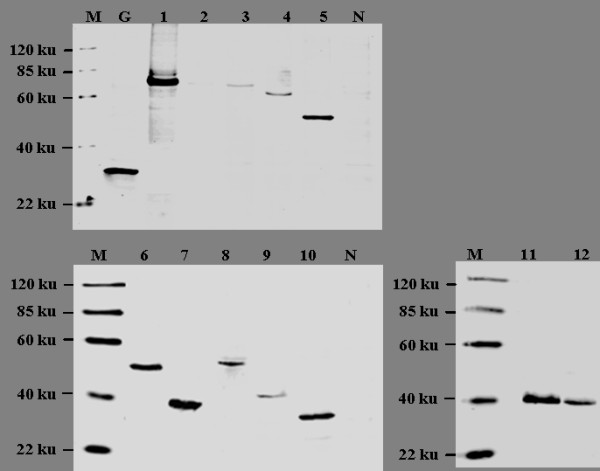
**Detection of the protein expressed by 12 subcloning cDNA of CSFV NS2 gene**. M, Protein Marker; N, negative control; 1~12, Proteins encoded by 12 sub-cloning cDNA of CSFV NS2 gene

**Figure 5:**
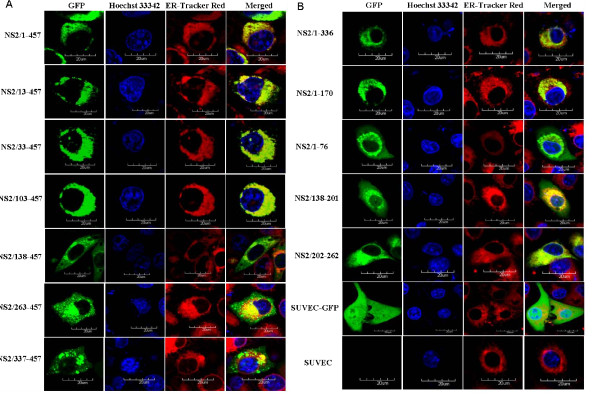
**Detection of the subcellular localization of the protein expressed by 12 subcloning cDNA of CSFV NS2 gene (A and B). Confocal microscopy images of SUVEC cells**. All the cells were stained by Hoechst33342 and ER-Tracker Red. Merged images show co-localization of fusion protein, GFP and the negative control. Bar = 20 μm for all the figures.

Western blot analysis showed that all the target protein were correctly expressed and displayed the expected molecular weight (Figure 4). The subcellular localization of NS2 was investigated by confocal fluorescence microscopy. The subcloned proteins NS2/1-457, NS2/13-457, NS2/33-457, NS2/103-457, NS2/1-336, NS2/1-170 and NS2/202-262 localized in the ER, whereas, proteins NS2/138-457, NS2/263-457, NS2/1-76 and NS2/337-457 were distributed in the cytoplasm, gathering around the ER. Protein NS2/138-201 was observed in the nucleus and cytoplasm, with no obvious gathering around the ER. In the GFP positive control cells, GFP was observed in the nucleus and cytoplasm (SUVEC-GFP), and no green fluorescence was detected in the negative control SUVECs (Figure 5A and 5B).

### NS2 contains two internal signal sequences and multiple domains in the ER

**Figure 6:**
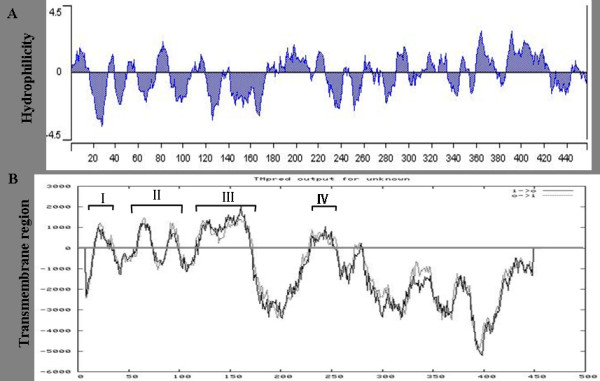
**Prediction of hydrophilicity region and transmembrane region of CSFV NS2 protein (A and B)**. I, II, III, and IV indicate the four putative transmembrane domains.

**Figure 7:**
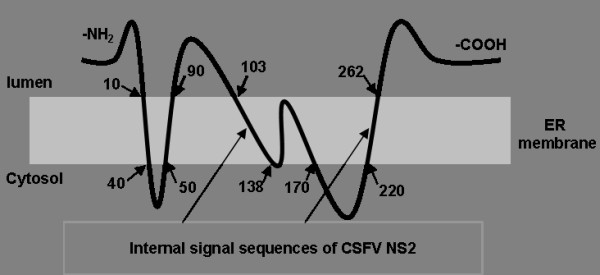
**Prediction of membrane topology of CSFV NS2 protein**. The numbers represent the amino acids identified by the deletion mapping experiments. Arrows show the locations of the two internal signal sequences.

Hydrophilicity analysis of the CSFV NS2 amino acid sequences using the DNAstar software showed that the -NH_2 _terminal was highly hydrophobic; accordingly, five probable transmembrane regions were predicted at the -NH_2 _terminal of the NS2 protein using the online tool available at http://us.expasy.org/tools (Figure 6A and 6B). Taken together with the data regarding the subcellular localization of 12 the subcloned NS2 fragments, the possible locations of the transmembrane domains were predicted. Thus, four transmembrane domains were predicted to reside within amino acids 1-40, 50-90, 103-170 and 220-262, with two internal signal sequences likely residing within amino acids 103-138 and 220-262. The predicted model of CSFV NS2 membrane topology is show in Figure 7.

## Discussion

CSF caused by virulent strains of CSFV is a hemorrhagic disease of pigs, characterized by disseminated intravascular coagulation, thrombocytopenia and immunosuppression. Recently, many researchers have focused on the development of novel vaccine and diagnostic methods, however, the molecular pathogenesis of CSFV is still not well understood. Regarding the function of virus-encoded proteins, Npro, NS3 and NS5B proteins have been studied extensively. However, NS2 protein was thought to function only as an NS2/NS3 auto-protease essential for the high productivity of CSFV in vivo [1,3,5,6]. To date, no reports have focused on the subcellular localization of NS2 protein.

Previously, researchers only speculated that CSFV NS2 protein was associated with the membrane, and it's translocation depended on p7 peptide; however, there was no experimental data that demonstrated the molecular mechanism behind it's subcellular localization. Our previous work demonstrated that, CSFV NS2 protein localized in the ER of host cells. The protein tag green fluorescence protein (GFP) was expressed as a fusion with CSFV NS2 protein and ER localization of the fusion protein showed no cell type specificity, and whether GFP was fused to the -COOH terminal or the -NH2 terminal did not effect translocalization of CSFV NS2 protein [7]. To reveal the molecular mechanism of ER localization of NS2 protein, the amino acid sequences of NS2 was analyzed using bioinformatics tools. The results indicated that the -NH2 terminal is highly hydrophobic, containing at least four transmembrane regions. Twelve subcloned cDNAs of the NS2 gene were expressed as GFP fusion proteins and confocal microscopy observation suggested that the proteins lacking the -NH2 terminal (NS2/337-457) distributed in cytoplasm, implying that there was no internal signal sequence in the amino acids 337-457. Proteins NS2/201-262 and NS2/1-170 localized to the ER, and together with the bioinformatics data, this suggested there were internal signal sequences residing within amino acids 220-262 and 103-138. A model of CSFV NS2 membrane topology was predicted, as shown in Figure 7; however further experiments are needed to investigate the natural subcellular localization of NS2 protein in CSFV infected cells. In contrast, the deletion of the NS2 gene by PCR perhaps influences translocalization of the target protein. Truncation by PCR deletion may have disrupted the internal signal sequences of the NS2 protein. Therefore, membrane topology was predicted from confocal microscopy data and through bioinformatics analysis of NS2 amino acid sequences.

The biofunction of a protein is always associated with it's subcellular localization. Previous studies showed that HCV NS2 protein localized in the ER independently of p7 protein [8,10], induced ER stress of host cells and consequently played an important role not only in the regulation of the host cells physiological functions but also in the pathogenesis of HCV[11]. Interestingly, CSFV NS2 shares a high level of similarity with the HCV NS2 protein regarding subcellular localization and auto-protease activity[5,12-14]. Both were able to induce ER stress and inhibit the proliferation of the host cells[7,15], and were essential for the production of infectious viral particles [6,16-18]. Therefore, we speculate that CSFV NS2 protein also plays an important role in the pathogenesis of CSF. The findings of this study provided a foundation for further work to reveal the biofunction of NS2 protein.

## Competing interests

The authors declare that they have no competing interests.

## Authors' contributions

KKG and QHT planned and participated in all of the experiments and wrote the manuscript. YMZ designed the project. KK participated in the confocal microscopy; LH contributed to cell culturing, plasmid construction and cell transfection. All authors have read and approved the final manuscript.

## References

[B1] Lackner T Muller A Pankraz A Becher P Thiel HJ Gorbalenya AE Tautz N Temporal modulation of an autoprotease is crucial for replication and pathogenicity of an RNA virus J Virol 2004 78 10765 10775 10.1128/JVI.78.19.10765-10775.2004 15367643 PMC516412

[B2] Harada T Tautz N Thiel HJ E2-p7 region of the bovine viral diarrhea virus polyprotein: processing and functional studies J Virol 2000 74 9498 9506 10.1128/JVI.74.20.9498-9506.2000 11000219 PMC112379

[B3] Agapov EV Murray CL Frolov I Qu L Myers TM Rice CM Uncleaved NS2-3 is required for production of infectious bovine viral diarrhea virus J Virol 2004 78 2414 2425 10.1128/JVI.78.5.2414-2425.2004 14963137 PMC369244

[B4] Elbers K Tautz N Becher P Stoll D Rumenapf T Thiel HJ Processing in the pestivirus E2-NS2 region: identification of proteins p7 and E2p7 J Virol 1996 70 4131 4135 8648755 10.1128/jvi.70.6.4131-4135.1996 PMC190302

[B5] Lackner T Thiel HJ Tautz N Dissection of a viral autoprotease elucidates a function of a cellular chaperone in proteolysis Proc Natl Acad Sci USA 2006 103 1510 1515 10.1073/pnas.0508247103 16432213 PMC1360547

[B6] Moulin HR Seuberlich T Bauhofer O Bennett LC Tratschin JD Hofmann MA Ruggli N Nonstructural proteins NS2-3 and NS4A of classical swine fever virus: essential features for infectious particle formation Virology 2007 365 376 389 10.1016/j.virol.2007.03.056 17482232

[B7] Tang QH Zhang YM Fan L Tong G He L Dai C Classic swine fever virus NS2 protein leads to the induction of cell cycle arrest at S-phase and endoplasmic reticulum stress Virology Journal 2010 7 4 10.1186/1743-422X-7-4 20064240 PMC2819037

[B8] Yamaga AK Ou JH Membrane topology of the hepatitis C virus NS2 protein J Biol Chem 2002 277 33228 33234 10.1074/jbc.M202304200 12082096

[B9] Hong HX Zhang YM Xu H Su ZY Sun P Immortalization of swine umbilical vein endothelial cells with human telomerase reverse transcriptase Mol Cells 2007 24 358 363 18182851

[B10] Lorenz IC Marcotrigiano J Dentzer TG Rice CM Structure of the catalytic domain of the hepatitis C virus NS2-3 protease Nature 2006 442 831 835 10.1038/nature04975 16862121

[B11] Moradpour D Penin F Rice CM Replication of hepatitis C virus Nat Rev Microbiol 2007 5 453 463 10.1038/nrmicro1645 17487147

[B12] Schregel V Jacobi S Penin F Tautz N Hepatitis C virus NS2 is a protease stimulated by cofactor domains in NS3 Proc Natl Acad Sci USA 2009 106 5342 5347 10.1073/pnas.0810950106 19282477 PMC2663979

[B13] Pieroni L Santolini E Fipaldini C Pacini L Migliaccio G LaMonica N In vitro study of the NS2-3 protease of hepatitis C virus Journal of Virology 1997 71 6373 6380 9261354 10.1128/jvi.71.9.6373-6380.1997 PMC191910

[B14] Welbourn S Pause A The hepatitis C virus NS2/3 protease Curr Issues Mol Biol 2007 9 63 69 17263146

[B15] Yang XJ Liu J Ye L Liao QJ Wu JG Gao JR She YL Wu ZH Ye LB HCV NS2 protein inhibits cell proliferation and induces cell cycle arrest in the S-phase in mammalian cells through down-regulation of cyclin A expression Virus Res 2006 121 134 143 10.1016/j.virusres.2006.02.004 16797769

[B16] Jones CT Murray CL Eastman DK Tassello J Rice CM Hepatitis C virus p7 and NS2 proteins are essential for production of infectious virus J Virol 2007 81 8374 8383 10.1128/JVI.00690-07 17537845 PMC1951341

[B17] Murray CL Jones CT Rice CM Architects of assembly: roles of Flaviviridae non-structural proteins in virion morphogenesis Nat Rev Microbiol 2008 6 699 708 10.1038/nrmicro1928 18587411 PMC2764292

[B18] Yi M Ma Y Yates J Lemon SM Trans-complementation of an NS2 defect in a late step in hepatitis C virus (HCV) particle assembly and maturation PLoS Pathog 2009 5 e1000403 10.1371/journal.ppat.1000403 19412343 PMC2669722

